# Biosynthesis of copper nanoparticles using *Alstonia scholaris* leaves and its antimicrobial studies

**DOI:** 10.1038/s41598-024-56052-y

**Published:** 2024-03-07

**Authors:** Ahmad Nasir Labaran, Zakariyya Uba Zango, Giriraj Tailor, Ahmed Alsadig, Fahad Usman, Muhammad Tukur Mukhtar, Alhassan Muhammad Garba, Raed Alhathlool, Khalid Hassan Ibnaouf, Osamah A. Aldaghri

**Affiliations:** 1 Department of Chemistry, Faculty of Science and Technology, Mewar University, Gangrar, Chittorgarh, Rajasthan 312901 India; 2 Department of Chemistry, College of Natural and Applied Science, Al-Qalam University, Katsina, Nigeria; 3 CNR NANOTEC Institute of Nanotechnology, Via Monteroni, 73100 Lecce, Italy; 4 Engineering Unit, Department of Mathematics, Connecticut State Community College Norwalk, Connecticut State Colleges and Universities (CSCU), Hartford, USA; 5 Department of Physics, College of Science, Imam Mohammad Ibn Saud Islamic University (IMSIU), 13318 Riyadh, Saudi Arabia

**Keywords:** *Alstonia scholaris*, Antimicrobial, Copper nanoparticles, Characterization, Extract, Chemistry, Materials science

## Abstract

The utilization of plants for the production of metallic nanoparticles is gaining significant attention in research. In this study, we conducted phytochemical screening of Alstonia scholaris (*A. scholaris*) leaves extracts using various solvents, including chloroform, ethyl acetate, n-hexane, methanol, and water. Our findings revealed higher proportions of flavonoids and alkaloids in both solvents compared to other phytochemical species. In the methanol, extract proteins, anthraquinone and reducing sugar were not detected. On the other hand, the aqueous extract demonstrated the presence of amino acids, reducing sugar, phenolic compounds, anthraquinone, and saponins. Notably, ethyl acetate and chloroform extracts displayed the highest levels of bioactive compounds among all solvents. Intrigued by these results, we proceeded to investigate the antibacterial properties of the leaf extracts against two major bacterial strains, Escherichia coli (*E. coli*) and Staphylococcus aureus (*S. aureus*). All extracts exhibited significant zones of inhibition against both bacterial isolates, with *S. aureus* showing higher susceptibility compared to *E. coli*. Notably, the methanol extract displayed the most potent I hibitory effect against all organisms. Inspired by the bioactivity of the methanol extract, we employed it as a plant-based material for the green synthesis of copper nanoparticles (Cu-NPs). The synthesized Cu-NPs were characterized using Fourier infrared spectroscopy (FT-IR), UV–visible spectroscopic analysis, and scanning electron microscopy (SEM). The observed color changes confirmed the successful formation of Cu-NPs, while the FTIR analysis matched previously reported peaks, further verifying the synthesis. The SEM micrographs indicated the irregular shapes of the surface particles. From the result obtained by energy dispersive X-ray spectroscopic analysis, Cu has the highest relative abundance of 67.41 wt%. Confirming the purity of the Cu-NPs colloid. These findings contribute to the growing field of eco-friendly nanotechnology and emphasize the significance of plant-mediated approaches in nanomaterial synthesis and biomedical applications.

## Introduction

Nowadays, incorporating green chemistry into nanoparticle research is a key area of development in material science and engineering^1,2^. Undoubtedly, the rise in demand of fabricating nanoparticle using straightforward, economic, and non-toxic routes, have been vital in overcoming the adverse effects of the existing sources, especially for clinical and catalytic applications^3,4^. In this regard, the utility of plant-based extracts would be very desirable in the fabrication and architecture of nano-sized materials and other nanoparticle-embedded products as it brings a crucial synergy between nanotechnological tools and plant sciences^5^. Among various classes of nanomaterials, metallic nanoparticles have gained tremendous scientific interests because of their unique properties^6–8^. Many of their outstanding applications include catalysis^9,10^, biological labeling^11,12^, drugs delivery^13,14^, tissue engineering^15,16^, optoelectronics^17,18^ and wastewater remediation applications^19,20^. However, most reported synthetic approaches use organic solvents, hydrazine^21^, sodium borohydride^21^, as surfactants, stabilizers as well as the reducing agents^22^. These reagents are regarded as hazardous to the environment. In addition, most of the preparation methodologies necessitate intricate controls and conditions, leveling up the cost of production.

Today, biosynthesis approaches for metal nanoparticles production are tremendously followed and optimized as it does not involve the use of hazardous chemicals^23,24^. However, synthesis approach from extracts from plant sources confirmed the advantages of the method to the microbial sources, associated with economic burdens, and the need for skilled personnels^25^. The growth and development of microbes’ resistant antibiotics prompted the research on finding alternative materials from a variety of sources^26^. Researchers have explored the used of various alternatives from plants and animal sources to synthesize materials that can be use as remedies against diseases. Plant materials such as leaves, roots and herbs for antimicrobial^27^, anticancer^3^, antidiabetic^28^, and other biological applications^29^. Metal nanoparticles (MNPs) synthesized from plant materials have found greater applications in the medicinal field. They are widely employed for the treatment of diseases. Among the various MONPs, Copper nanoparticles (CuNPs) has been widely investigated. It has shown activity against diseases as anti-inflammatory, anti-viral, and antioxidant. The properties of the CuNPs make it a promising candidate for use in medical applications such as wound healing, drug delivery, and cancer therapy. Furthermore, it has been relatively non-toxic against mammalian cells even at lower concentrations, making it ideal option for use in biomedical applications.

Among many potential medicinal plants, *Alstonia scholaris *(*A. scholaris*) commonly known as blackboard tree or devil tree is a native plant species found in various countries such as India (locally called saptaparni) Cambodia (called popeal khe), China (locally referred as Tang jiao shu) Indonesia (pulai as popularly called) Laos (called as dtin pet) in Malaysia (Popularly called Rejang or Pulai) Myanmar (Toungmayobeng s the local name), Philippines (Ditaa popularly called), and Vietnam (Suȧ, mo cua as the local name)^30,31^. It is a member of Apocyanacae that contains various metabolites such as monoterpenoid and indole alkaloids. It is described as a sinister tree, growing up to a to a height of 18 m and mostly founds n the forests of India, Pacific Asia, Queensland as well as South China. According to a myth of Hindu, the tree is an abode of witches and source of evil spirits^32,33^. It is widely used for herbal medication against various diseases such as malaria, fever, gastrointestinal disorders, ulcer dysentery, diarrhea, inflammation, cardiac and respiratory ailments. It is also used for the treatment of cancer^34^.

Despite various phytochemical screening research have indicated the antimicrobial activity of the *A. scholaris*, further studies regarding identification of its potentiality is essential for its usage, especially for the antimicrobial effects. Given their catalytic, plasmonic, magnetic, and thermal properties, Copper nanoparticles (Cu-NPs) gained particular interest for various applications^35^. Cu-NPs also spark fascinating research activity up to the high expectations it generates, especially in biochemistry. A key advantage of Cu is its affordability and availability, rendering the production of CuNPs cost-effective. So far, CuNPs have been synthesized from several plant extracts^11,36^. However, the potentiality of using *A. scholaris* extracts is still not yet explored.

**Figure 1:**
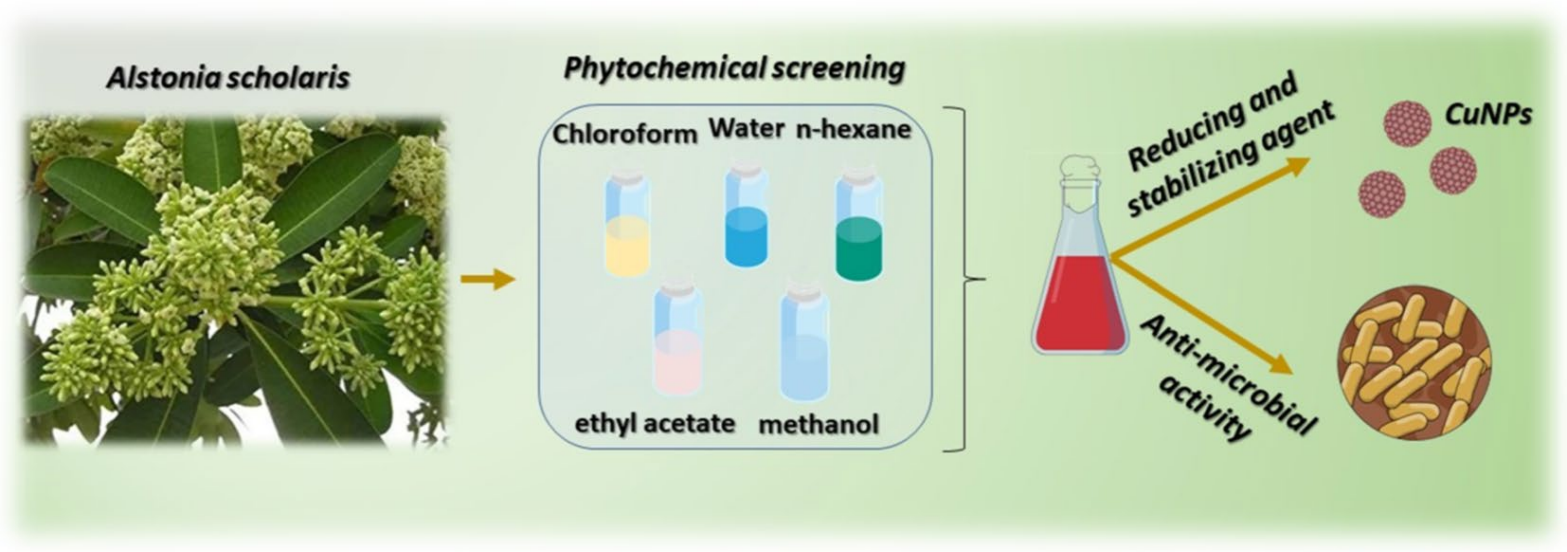
Schematic diagram, presenting the general aim of the research findings.

Herein, we aimed at investigating the effects of solvents extracts in phytochemical screening of the *A. scholaris* and determination of its antimicrobial activities (Fig. 1). Considering the green synthesis benefits of Cu-NPs and their potentials in the antimicrobial studies, we also investigated the usability of *A. scholaris* extracts as an alternative bio-source for reducing Cu ions to form Cu-NPs. The *A. scholaris* extracts was chosen due to its relative content of as previously reported. The as-made NPs are characterized thoroughly to determine the properties of the synthesized nanostructures. The Cu-NPs have found versatile applications in biomedicals, environmental and other potential applications.

## Materials and methods

### Collection of plant leave and preparation of aqueous and organic extract

The leaves of *Alstonia scholaris* was collected from the depository/herbarium of the Mewar University, Chittorgarh Rajasthan India. The experiments were performed according to the guidelines and legislation of the Indian Institute of Interrogative Medicine.

To prepare the extract, the fresh piece of *A. scholaris* leaves was thoroughly washed using tap water, then with distilled water for the dust removal. It was then cut and set for drying at ambient temperature for a period of five days. The dried leaves were then grinded using a sterile blender, and about 40 g was weighed and subjected to 800 °C boiling in a distilled water at (400 mL) for the duration of 15 min. After cooling down, the sample was filtered (using Muslin paper) to get the required amount^37^. 250 mL Erlenmeyer flasks was used for the extract collection and kept at 4 °C for later usage. Additional extracts were made in an identical fashion, except that the water was substituted with the selected solvents. For solvent extraction of phytochemicals *A. scholaris* leaves, the finely powdered leaves (100 g) were placed in a 500 mL Soxhlet apparatus and various solvents such as chloroform, ethyl acetate, n-hexane, methanol as well as aqueous solvents were used for the extraction for 24 h in each case.

### Phytochemical screening of crude extracts

Phytochemical screening was conducted to test for chemical constituents such as reducing sugar, steroid, glycoside, flavonoids, alkaloids, saponins, phenolic compounds/ tannins, anthraquinone, proteins, and amino acids. Thus, the analysis for determining these compounds in the leaves sample of the *A. scholaris* is in the following sections. To carry out the analysis of steroids and terpenes (referred to as Salkowski test), 0.5 g of the sample in a test tube (20 mL) was used. 1 mL of the chloroform as a solvent was introduced, then dropwise of concentrated sulfuric acid (1 mL), resulting in two phases partitioning. The yellowish precipitate formation conclude the likelihood of steroids/terpenes in the sample^38^. The screening for chemical composition has been carried out according to the previous studies reported for flavonoids^38^, protein^39^, amino acid testing^39^, reducing sugars testing^39^, tannins^38^, anthraquinones^38^, saponins^39^, and glycosides^40^.

### Antimicrobial activity of crude extracts

All procedures were carried out aseptically. The pathogenic strain of *E. coli* and *S. aureus* were sourced from the laboratory at the Life Sciences Department of the University of Mewar. The medium was made by adding 28 g of nutrient agar in distilled water of 1000 mL. Using hot magnetic stirrer, this substance was heated to fully dissolve the metal. The dissolved media was autoclaved for 15 min at 121 °C and 15 psi. Following autoclaving, the sterilized medium made of 15 mL distributed into the Petri plate and set to crystallized for culturing. The pathogenic microorganism, (*E. coli* and *S. aureus)* were subculture on the media and put in an incubator incubated at 37 °C.

In sterilized Glass bottles, different concentration of the extracts was made by weighing 40, 20, 10, 5, 2.5 and 1.5 mg of each of the extract into 1 mL of 10% DMSO for a better stability of the extract. It was then covered with foil paper. The antimicrobial activity of the extracts was analyzed against the strains of the bacteria *E. coli* (the Gram-negative) and *S. aureus* (the Gram-positive) via diffusion technique. A colony of 1000,000 forming units (CFU) of the microorganisms (*E. coli* and *S. aureus)* were inoculated on the plate. At the prepared concentrations (40, 20, 10, 5, 2.5 and 1.25 mg/mL) each extract was introduced to the nutrient agar plate containing the bacteria strains. The plates were subjected to incubation at 37 °C for a night. After the period of incubation, each of the plates were analyzed for the zone of inhibition. The antibacterial activity of prepared extracts was measured by the zones of inhibition after the incubation period (24 h) at temperature of 37 °C.

### Synthesis and characterization copper nanoparticles (Cu-NPs)

The green synthesis of Cu-NPs was carried out. For the procedure, the *A. scholaris* leaves broth of known concentration (10 mL) was mixed with 0.1 M CuSO_4_ solution by mixing at a specific ratio (1:9). Gradual transformation of mixture from light blue to light green to dark green indicated the formation of copper nanoparticles^41^.

Absorption of ultraviolet (UV) and visible radiation is one of the most routinely used analytical tools in life sciences research. The simplest application of UV/Visible radiation is used to quantify the amount of a substance present in a solution. Thus, the as-synthesized Cu-NPs was characterized by UV–vis spectrophotometer recorded at Lambda max 750 (Perkin Elmer)^42^.

Fourier Transformed Infra-red (FT-IR) spectroscopy was used to identify the functional groups that are bound to the surface of nanoparticles and that lead to the stability of the material. The identification of the active functional group is important in developing new approaches in the synthesis of the CuNPs. Thus, the functional groups identification was achieved using PerkinElmer FTIR spectrometer. The sample was prepared using KBr pellets and scanned from 500 to 4000 cm^−1^ for the finger and functional group regions^42^.

Scanning electron microscopy (SEM) are used to show the morphology and the shape of the synthesized nanoparticles, SEM (Nova Nano SEM 450 (dwell 10 µs)) was used. For this purpose, different images at various magnifications are taken from the sample. Similarly, to analyse the surface characteristics as well as elemental compositions of the material, the Energy Dispersive X-ray spectroscopy (EDX) was used. Each element has a unique atomic structure making a unique set of peaks on its X-ray spectrum which, in turn, leads to the characterization of the elements^43^.

### Statistical analysis

All the experiments were performed in triplicates. The values obtained were represented by a mean ± standard error. The data obtained were statistically analyzed excel.

## Results and discussion

**Figure 2:**
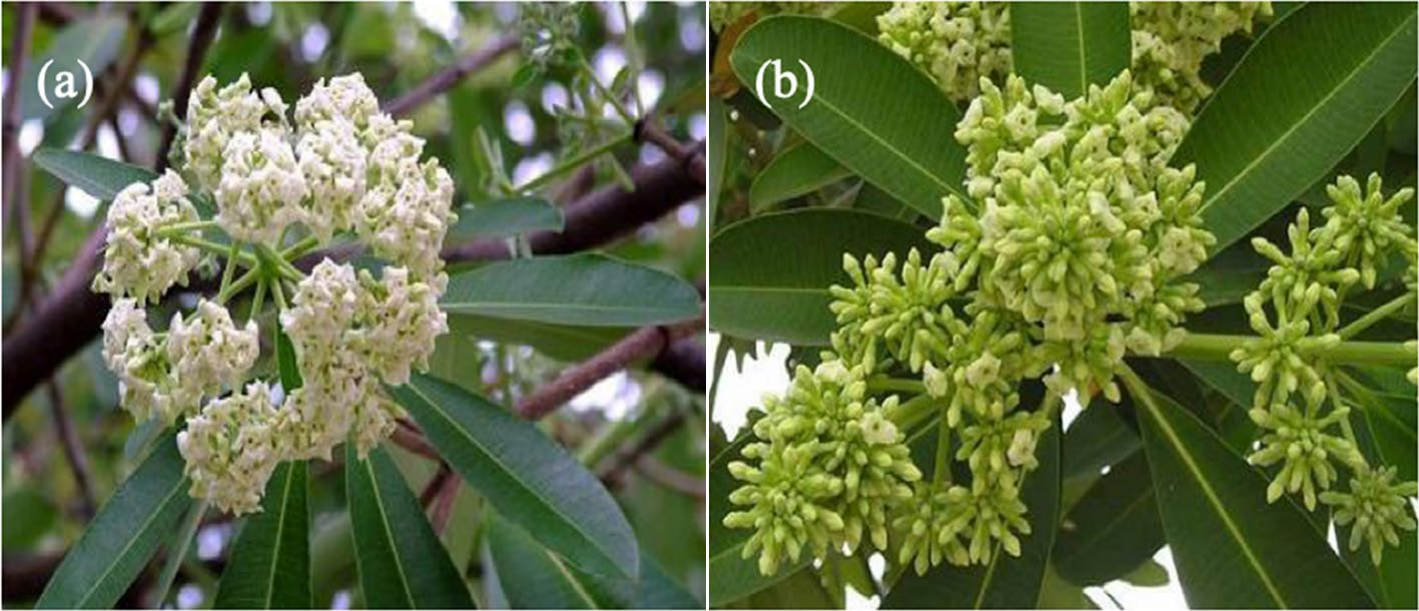
Digital photographs of (**a**) image of tree and (**b**) image of *A scholaris leaves*.

Figure 2 depicted the tree and the leaves of the *A. scholaris*. The bark is of the tree is dark-brown in color with smooth texture and milky latex^32^.The leaves are non-stipules, with about 5–8 whorls. The petiole has a width of about 2 cm diameter and stout and flowers are ephemeral, with axillary cymes arrangement made of greenish–white color^33^. The leaf is made up of medium to large size dorsiventral blade. The venation of the leaf is pinnate, consisting of large number of veins^44^.

The result of phytochemical analysis of the *A. scholaris* leave extracts in the various solvents are presented in Table 1. It has shown flavonoids and alkaloids presence at higher proportions, in both solvents in comparison to other phytochemicals. The methanol extract has shown the presence of all metabolites, with exception of proteins, anthraquinone and reducing sugar. The aqueous extract contained reducing sugar, amino acid, phenolic compounds, anthraquinone and saponins. Of all, ethyl acetate and chloroform contained the lowest amount of the bioactive molecules. According to Anthony et al., the extracts of the leave contained the most essential compounds in the ethyl acetate, butanol and water as the extracting solvents^39^. Additionally, higher proportions are present in the bark and leaves of the aqueous. The most detected ones where; proteins, steroids, phenols and tannins^39^**.** In a qualitative comparative review presented by Khyade et al., for the phytochemical screening of *A. scholaris* leaves, most of the studies conducted have majorly reported carbohydrates, oils and fats alkaloids, flavonoids, tannins, terpenoids, saponins and steroids. For instance, Misra et al., reported that extract of n-hexane has alkaloids and steroids as metabolites. However, the isopropanol extract of the leave has shown the terpenoids in smaller quantity in comparison to root Mistry and bark of the plant. Mistry and Parekh was observed oils and fats, alkaloids, flavonoids, terpenoids, glycosides, saponins, mucilage and gums in both bark, leaves and stem of the plant^45^.

**Table 1:** Screening of phytochemical in the extracts of *A. scholaris*.

Analytical parameters	n-hexane	Chloroform	Ethyl acetate	Methanol	Water
Steroids	−	−	+	+	−
Alkaloids	+	+	−	−	−
Dragendoff test	+	+	+	+	+
Mayer’s test	−	−	−	−	−
Flavonoids	+	+	+	+	+
Protein	−	−	−	−	−
Amino acid	−	−	−	+	+
Reducing sugar	−	−	−	−	+
Tannins	−	−	−	+	+
Anthraquinone	+	+	+	−	+
Saponins	−	−	+	+	+
Glycosides	+	+	−	+	−

Key: (+) indicates presence, while (−) indicates absence.

Thus, we analyzed the antimicrobial properties of the *A. scholaris* plant. The standard antibiotics used as a standard control in antibacterial activity test was Gentamicin GEN, Ciprofloxacin (CPR), Oploxacin (OFL), Augmentin (AUG), and Nitrofurantoin (NIT). The results from the assessment of the antibacterial properties *E. coli* and *S. aureus* has been determined. It has shown the effects of extracts on the chosen bacterial pathogens under the zone of inhibitory study.

Tables 2, 3, 4, and 5 have shown the antibacterial activities on the crude extracts. The results revealed the excellent inhibition against the growth of all the bacteria tested at predetermined studied concentrations. For n-hexane (Table 2), chloroform (Table 3), ethyl acetate (Table 4) and methanol (Table 5) extracts, *S. aureus* has the highest activity while *E. coli* has the lowest. Methanol extract also demonstrated the highest inhibitory effect for the studied organisms. Previous finding from Thankamani et al.^46^, revealed the effect of extract of methanol which possessed the highest inhibition against the microorganism and was more pronounced at the inhibitory zone of 15–25 mm. Whereas chloroform extract has less inhibition effect for the *S. aureus*. Accordingly, the inhibitory effect of n-hexane and ethyl acetate extract was closely the same with only slight variation for the studied organisms. Study conducted by Anthony et al.^47^, reported that methanol extracts of *A. scholaris* leaf exhibited much significant antibacterial activities in comparison to other extracts employed.

**Table 2:** Antibacterial effect of extract of n-hexane for *A. scholaris* leave against the tested bacteria.

Microorganisms	Concentration (mg/mL)					
	40	20	10	5	2.5	1.25
Zone of inhibition (mm)						
* E. coli*	6	4	3	2	2	2
* S. aureus*	7	6	6	4	3	3

**Table 3:** Antibacterial activities of extract of chloroform for *A. scholaris* leave against the tested bacteria.

Microorganisms	Concentration (mg/mL)					
	40	20	10	5	2.5	1.25
Zone of inhibition (mm)						
* E. coli*	15	12.5	10	8	5	3
* S. aureus*	13	13	12	10	8	5

**Table 4:** Antibacterial effect of extract of ethyl acetate for *A. scholaris* leave against the tested bacteria.

Microorganisms	Concentration (mg/mL)					
	40	20	10	5	2.5	1.25
Zone of inhibition (mm)						
*E. coli*	10	9	7	5	3	1
*S. aureus*	9	8	6	4	3	2

**Table 5:** Antibacterial effect of extract of methanol for *A. scholaris* leave against the tested bacteria.

Microorganisms	Concentration (mg/mL)					
	40	20	10	5	2.5	1.25
Zone of inhibition (mm)						
*E. coli*	23	21	20	17	13	9
*S. aureus*	21.5	20	19.5	18	15	11

Biological route for the nanoparticle’s synthesis using plant materials has a lot of advantages over synthesis techniques. It is ideal for a large-scale synthesis of the nanoparticles.

**Figure 3:**
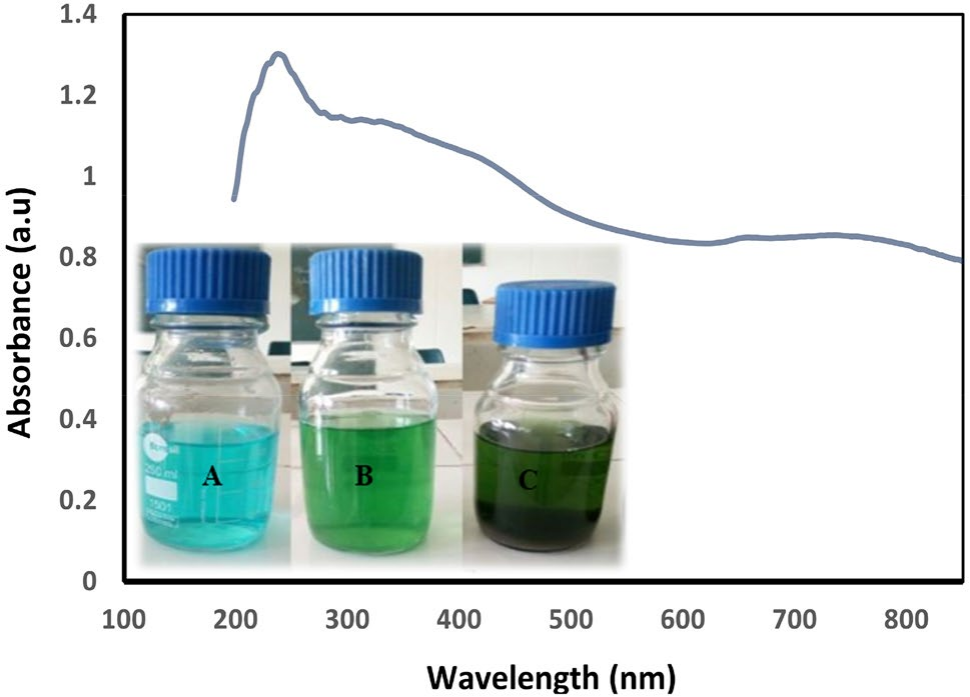
Optical profile of CuNPs produced using *A. scholaris* leaf aqueous extract. Color changes during synthesis of Cu-NPs (**A**–**C**), which indicates Cu-NPs formation.

The biosynthetic route for the synthesis of copper nanoparticles (Cu-NPs) using plant extracts, such as A. scholaris, is considered suitable and economical for various applications in environmental, medical, and industrial fields. The biosynthetic route is suitable and economical for the Cu-NPs synthesis for environmental, medical, as well as industrial applications. In addition, it eliminates the use of toxic chemical substances which are potentially hazardous to the living organisms and the surrounding environments. Thus, an aqueous extract of the *A. scholaris* leave was employed for the Cu-NPs biosynthesis. The Cu-NPs was confirmly synthesized by the color changes reacting species according to the UV–visible spectroscopy^48^. Addition of the leaf broth to the CuSO_4_.5H_2_O solution, resulted in the color changes from light to dark green, confirming the successful formation of the Cu-NPs (Fig. 2). In a previously study, the formation of the dark green color which eventually changed to dark brown, reaffirmed the successful biosynthesis of the Cu-NPs. Also, the color change was attributed to the excitation resulting from surface Plasmon resonance (SPR)^49^. The spectrum was determined at 200–800 nm wavelength range (Fig. 3). The spectrum from UV–vis has 243 nm as the stable wavelength for the formation of Cu-NPs^50^.

**Figure 4:**
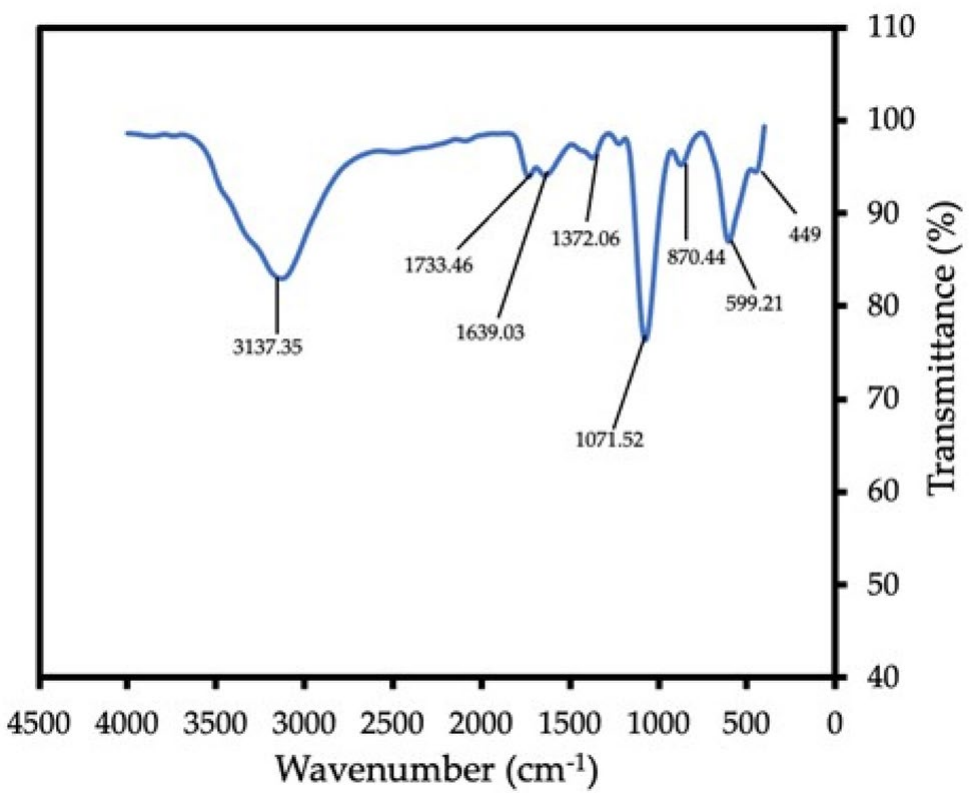
FTIR spectrum of Cu-NPs synthesized from *A. scholaris*.

The FTIR spectrum revealed the various functionalities in the Cu-NPs. It revealed various interactions as well as compositions of the leave extract (Fig. 4). The presence of broad peak at 3137.35 cm^−1^ highlighted the stretching due O–H from the alcohols and carboxylic acid present in the compounds^10,51^. The intense peak appeared around 1733.46 cm^−1^ represents C=O bending vibrations. Also, appearance of the IR peak at 1639.03 cm^−1^ shows the stretching occurred for C=C in the compounds. The peak at 1372.06 cm^−1^ was due to S =O vibration, while the peak at 1071.52 cm^−1^ indicated the stretching due to C–O band of the primary alcohols. Peak at 870.44 cm^−1^ shows C–H bending in the molecules, while the peak at 599.21 cm^−1^ was a result of C–I vibration of the halo species. The occurring of peak around 449 cm^−1^ corresponding on the of Cu ion specie. These peaks obtained were similar to those previously recorded in the literature such as the work of Thiruvengadam et al.^44^ and Rajeshkumar and Rinitha^52^. The FT-IR spectrum confirmed the possibility of the various interactions between the copper ion and the various functional groups contained in the extract^44^. Thiruvengadam et al.^50^, stated that the interaction between the Cu-NPs and the metabolites in the *A. scholaris* resulted in the possible reduction the NPs to copper as confirmed by the FTIR result.

**Figure 5:**
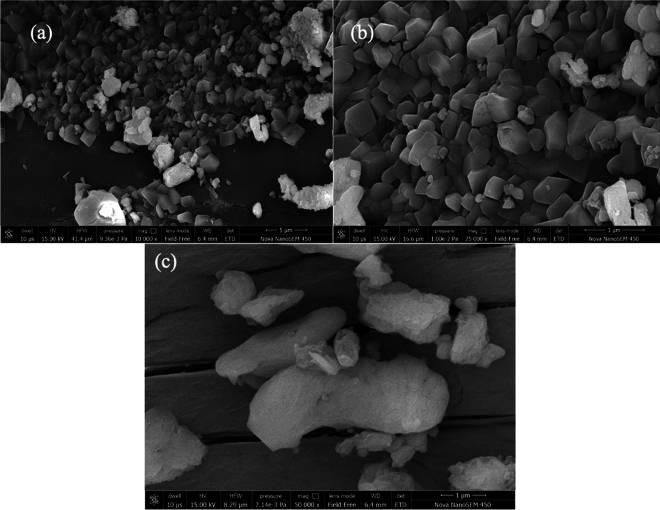
SEM micrographs of CuNPs at (**a**) 10 K, (**b**) 25 K and (**c**) 50 K magnifications.

The diverse shapes of the surface particles of the synthesized CuNPs, as observed in the SEM images in Fig. 5, suggest that the nanoparticles have a complex and heterogeneous structure. The presence of triangular, cylindrical, polygonal, and nearly spherical shapes indicates that the CuNPs have different facets and orientations, which may influence their physical and chemical properties. The irregular shapes of the CuNPs could be attributed to the synthesis method used, as well as the reaction conditions and parameters. Similar outcomes were reported, in which, SEM images of the synthesized Cu-NPs contained irregular particles arranged in clusters comprising of different structures from polygons to spherical shapes^50^. The unique shapes of the CuNPs could also have implications for their applications in various fields, such as catalysis, electronics, and biomedicine. Different shapes of nanoparticles have been shown to exhibit different catalytic activities, optical properties, and biological interactions. Therefore, understanding the morphology of the synthesized CuNPs is important for optimizing their performance in different applications. The results of the study suggest that A. scholaris, a plant species, can be effectively utilized for the biosynthesis of the Cu-NPs. The irregular shapes of the Cu-NPs synthesized using A. scholaris as observed in the SEM images indicate that the plant extract contains bioactive compounds that can effectively reduce and stabilize copper ions, leading to the formation of nanoparticles with diverse shapes.

**Figure 6:**
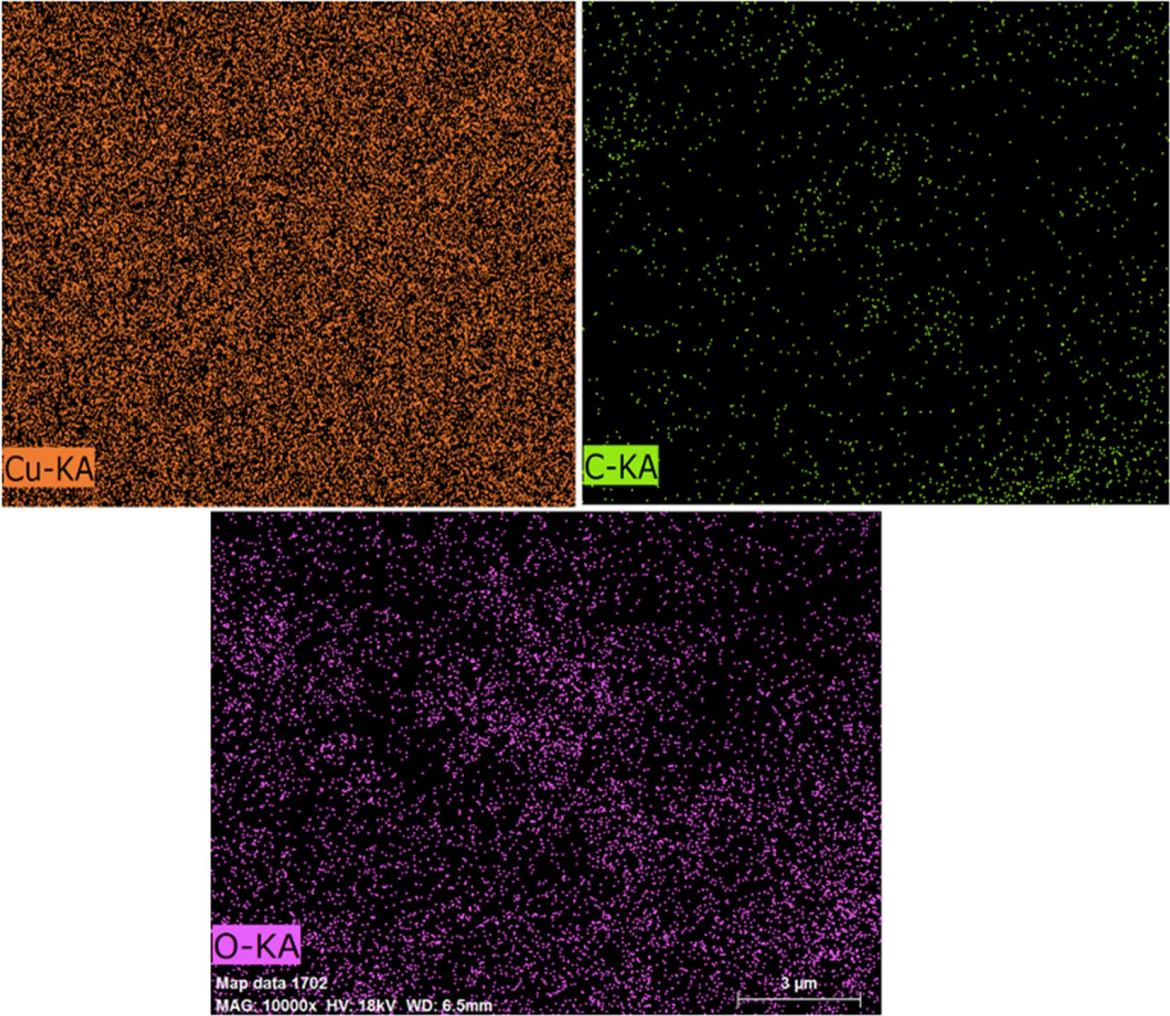
EDX mapping of the CuNPs.

**Figure 7:**
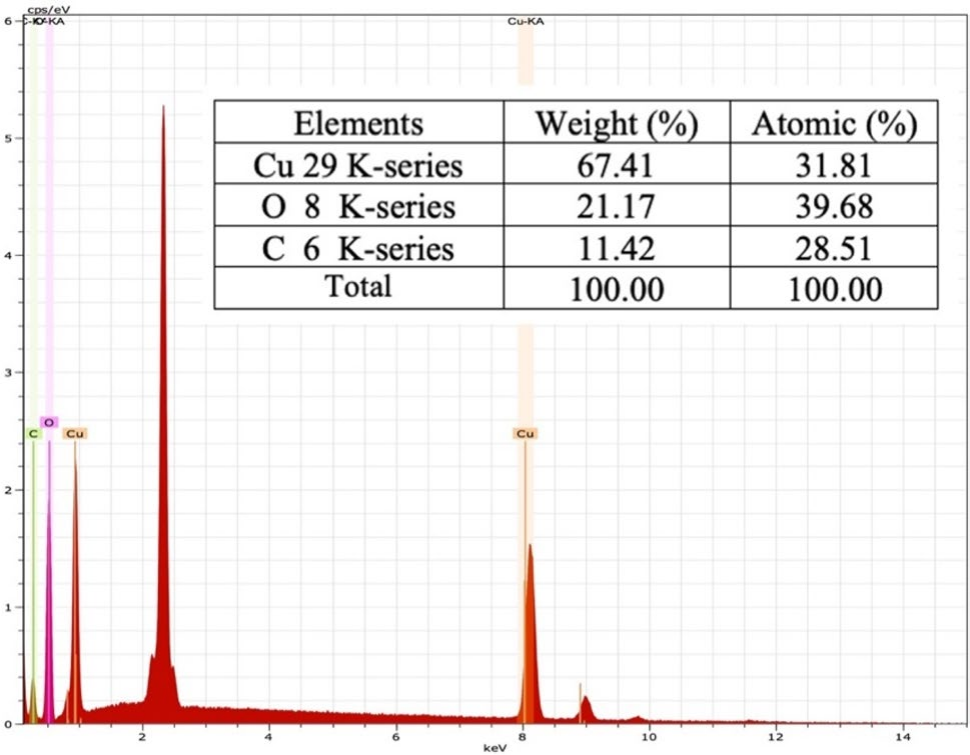
Cu-NPs Energy Dispersive X-Ray Analysis (EDX) spectrum.

The elemental composition revealed by the analysis provides valuable information about the chemical makeup of the Cu-NPs and can help in understanding their properties and potential applications. The EDX spectrum explained the elements composition on the surface of the Cu-NPs. The mapping result highlighted the major elements present **(**Fig. 6). The carbon and oxygen are appeared as the lowest peaks compared to the Cu. They resulted from the phytochemical metabolites present in leave extract^16^. Thus, they also served as the evidence for the for the reduction of Cu-NPs to Cu ions. The compositions of the elements from the EDX spectrum were depicted in Fig. 7. The presence of a prominent Cu peak in the elemental analysis indicates that the Cu-NPs synthesized have a high purity level of copper ions, with a relative abundance of 67.41%. This suggests that the majority of the elemental composition of the nanoparticles is copper, which is desirable for applications where high purity is important. It is then followed by the O and C with the relative abundance of 21.17 and 11.42%, respectively. This is contrary to the previous findings with much lower abundance of the Cu with the Cu content of 26.06% and 8.04%, respectively^48,52^. Thus, this result findings indicated that *A. scholaris* could be well utilized for the biosynthesis of Cu-NPs.

This green synthesis approach offers several advantages over conventional chemical methods, including eco-friendliness. The use of plant extracts for nanoparticle synthesis eliminates the need for toxic chemicals and reduces the generation of hazardous waste. This aligns with the principles of green chemistry and sustainable development. Additionally, the cost-effectiveness of the synthesis method is often more cost-effective compared to conventional chemical methods as they utilize natural resources that are readily available and relatively inexpensive. This makes the production of Cu-NPs more affordable and accessible. More so, the scalability allows to produce Cu-NPs in large quantities for industrial applications. This scalability is essential for commercialization and widespread use of the nanoparticles. Most importantly is the biocompatibility which makes it safe for use in medical applications, such as drug delivery, imaging, and therapy. The absence of toxic residues from the synthesis process enhances the biocompatibility of the nanoparticles.

## Conclusions

*A. scholaris* has been a widely known medicinal plant containing variety of bioactive compounds. In this work, leave extract of the *A. scholaris* has been analyzed for phytochemical composition and antimicrobial significance. The leave extracts have screened for various metabolites such as reducing sugar, amino acids, alkaloids, saponins, steroids tannins, phenolic glycosides, flavonoids, and anthraquinone. The antimicrobial activity of the extracts has been investigated against bacterial species, the *S. aureus* and *E. coli.* The extracts have shown significant zones of inhibition against the two bacterial isolates. *S. aureus* has shown an average highest activity while *E. coli* has shown an average lowest activity in all extracts. Methanol extract showed the highest inhibitory effect on all organisms. Biological synthesis of Cu-NPs has been investigated using the aqueous leave extract of the *A scholaris*. It has been well identified by UV–vis analysis, FT-IR and SEM (including the EDX). The Cu-NPs was confirmed formed by the color changes in from light to dark green. The FT-IR has revealed the major functional groups present in the *A. scholaris* as well as their chemical interaction. The images according to the SEM analysis revealed the irregular shapes the particles have consisting of triangular, cylindrical, polygonal spherical shapes. The EDX spectrum indicated the formation of the Cu-NPs based on the sharp and strong peak of the Cu ions.
